# Long Non-coding RNAs Are Central Regulators of the IL-1β-Induced Inflammatory Response in Normal and Idiopathic Pulmonary Lung Fibroblasts

**DOI:** 10.3389/fimmu.2018.02906

**Published:** 2018-12-11

**Authors:** Marina R. Hadjicharalambous, Benoit T. Roux, Carol A. Feghali-Bostwick, Lynne A. Murray, Deborah L. Clarke, Mark A. Lindsay

**Affiliations:** ^1^Department of Pharmacy and Pharmacology, University of Bath, Bath, United Kingdom; ^2^Division of Rheumatology and Immunology, Department of Medicine, Medical University of South Carolina, Charleston, SC, United States; ^3^RIA IMED Biotech Unit, AstraZeneca, Gothenberg, Sweden; ^4^MedImmune, Cambridge, United Kingdom

**Keywords:** idiopathic pulmonary fibrosis, long non-coding RNA, fibroblast, transcriptomics, inflammation, fibrosis, interleukin-1β

## Abstract

There is accumulating evidence to indicate that long non-coding RNAs (lncRNAs) are important regulators of the inflammatory response. In this report, we have employed next generation sequencing to identify 14 lncRNAs that are differentially expressed in human lung fibroblasts following the induction of inflammation using interleukin-1β (IL-1β). Knockdown of the two most highly expressed lncRNAs, IL7AS, and MIR3142HG, showed that IL7AS negatively regulated IL-6 release whilst MIR3142HG was a positive regulator of IL-8 and CCL2 release. Parallel studies in fibroblasts derived from patients with idiopathic pulmonary fibrosis showed similar increases in IL7AS levels, that also negatively regulate IL-6 release. In contrast, IL-1β-induced MIR3142HG expression, and its metabolism to miR-146a, was reduced by 4- and 9-fold in IPF fibroblasts, respectively. This correlated with a reduced expression of inflammatory mediators whilst MIR3142HG knockdown showed no effect upon IL-8 and CCL2 release. Pharmacological studies showed that IL-1β-induced IL7AS and MIR3142HG production and release of IL-6, IL-8, and CCL2 in both control and IPF fibroblasts were mediated via an NF-κB-mediated pathway. In summary, we have cataloged those lncRNAs that are differentially expressed following IL-1β-activation of human lung fibroblasts, shown that IL7AS and MIR3142HG regulate the inflammatory response and demonstrated that the reduced inflammatory response in IPF fibroblast is correlated with attenuated expression of MIR3142HG/miR-146a.

## Background

Interleukin-1β (IL-1β) is a potent pro-inflammatory mediator that is produced following activation of one of the multiple inflammasome multi-protein complexes. One of the best characterized is the nucleotide-binding oligomerization domain-like receptor (NLR) family, pyrin domain-containing 3 (NLRP3), whose activation and subsequent release of IL-1β, has been demonstrated in chronic obstructive pulmonary disease, severe asthma, and respiratory infections (1). Idiopathic pulmonary fibrosis (IPF) is a chronic disease characterized by scar tissue accumulation in the lungs leading to impaired gas exchange and restricted ventilation (2–4). The underlying causes of the disease are still unclear, although persistent epithelial injury and/or exposure to pathogens is thought to drive an exaggerated wound healing response from fibroblasts, that contributes toward the development and progression of IPF (2, 4). In the case of this respiratory disease, the role of IL-1β is yet to be established although there is a report of showing increased levels in bronchoalveolar lavage (5). In addition, there are conflicting reports as to whether IL-1β elicits pro-fibrotic or anti-fibrotic activities (6, 7). IL-1β was previously shown to drive IL-6 expression in orbital and synovial fibroblasts *in vitro* (8), although nothing is known regarding its effect on lung fibroblasts.

Non-coding RNAs (ncRNAs) are broadly classified as either short ncRNAs (<200 nucleotides) or long ncRNAs (>200 nucleotides). The microRNA (miRNA) family of short ncRNAs are the best characterized and known to induce mRNA degradation and/or suppress mRNA translation via the RNA interference pathway (9). There is now a considerable body of evidence to indicate that miRNAs are central regulators of the immune response (10). In particular, induction of miR-146a and miR-155 have been shown to be regulators of the inflammatory response in multiple cells types (11, 12). In contrast, much less is known about the function and mechanism of action of lncRNAs which are commonly grouped into long intergenic ncRNA (lincRNA) (located between protein coding genes), antisense (whose transcription overlaps protein coding genes on the opposite strand), and pseudogenes (non-translated versions of protein-coding genes) (13, 14). However, either through interactions with proteins and/or RNA/DNA pairing, there is now accumulating evidence to indicate that lncRNAs are novel regulators of multiple biological response, including inflammation (15, 16). Indeed, studies by ourselves and others have identified a number of lncRNAs that are differentially expressed following activation of innate immunity, which have been shown to regulate the subsequent inflammatory response including PACER (p50-associated COX-2 extragenic RNA) (17), THRIL (TNFα- and hnRNPL related immunoregulatory lincRNA) (18), lnc-IL7R (19), IL1β-RBT46 (20), lincRNA-COX2 (21, 22), lincRNA-EPS (23), lincRNA-Tnfaip3 (24), and IL7AS (25).

At the present time, nothing is known regarding the function of lncRNAs in the fibroblast inflammatory response and whether this is changed in IPF. In this report, we have employed next generation sequencing to examine the changes in the profile of lncRNA expression following the IL-1β-induced inflammatory response from human lung fibroblasts and determined whether these regulate this response in both control and IPF-derived fibroblasts.

## Materials and Methods

### Fibroblast Source and Cell Culture

Control (age = 50 ± 3 y; 3 male and 2 females) and IPF fibroblasts (age = 62 ± 1 y; 3 male and 2 females) were obtained from Professor Carol Ferghali-Bostwick (Medical University of South Carolina, USA) and the Coriell Institute of Medical Research (Camden, New Jersey, USA). Approval was obtained from the Medical Board of the Medical University of South Carolina and the Coriell Institute of Medical Research with patients proving material with informed consent. All methods were performed in accordance with the relevant guidelines and regulations. Neither the control or IPF patients had a history of smoking. Fibroblasts were cultured in DMEM (high glucose, pyruvate) growth media supplemented with 10% (v/v) FBS, 1% (v/v) Pen-Strep, and 0.1% (v/v) Fungizone. All cultures were maintained in a 37°C, 5% (v/v) CO_2_ humidified incubator. Upon reaching ~80–90% confluency cells were washed in sterile 1 × PBS followed by treatment with StemPro® Accutase® cell detachment solution. All experiments were performed using cells plated at passage 6–7.

### Preparation and Treatment of Fibroblasts Used in RNA-seq Study

Control lung fibroblasts (5 × 10^5^ cells) were seeded in 6-well cell culture plates (Corning Costar) on day 1 and left overnight. On day 2, the cells were serum-starved with 2 ml of fresh medium (0.1% FBS) and treated with/without 3 ng/ml IL-1β (recombinant, expressed in *E. coli*, Sigma-Aldrich, I9401-5UG) for 6 h before all supernatants were collected and cells were harvested for RNA extraction.

### Pharmacological Inhibition of Human IκB Kinase-2 (IKK-2)

Control and IPF fibroblasts (5 × 10^5^ cells) were seeded in 12-well cell culture plates on day 1 and left overnight. On day 2, the cells were serum-starved with 1 ml of fresh medium (0.1% FBS) and treated with/without 3 ng/ml IL-1β (recombinant, expressed in *E. coli*, Sigma-Aldrich, I9401-5UG) and TPCA-1 (0, 0.1, 1, 10 μM) (T1452, Sigma-Aldrich) for 24 h before supernatants were collected and cells were harvested for RNA extraction.

### RNA Isolation and Quality Control

RNA was extracted using the RNeasy kit (74104, Qiagen), including an on-column DNase treatment (79254, Qiagen) according to the manufacturer's guidelines. RNA concentration was determined using the Qubit 2.0 (Life Technologies). RNA quality was measured using the Agilent Bioanalyzer and produced RIN values of >8.0. When measuring miRNAs, total RNA was extracted using the miRNeasy Mini Kit (217004, Qiagen) with an on-column DNase treatment (Qiagen), according to the manufacturer's instructions.

### Transfection With LNA Antisense

On the day of transfection, 5 μL of HiPerFect (301705, Qiagen) was mixed with 200 μL of media without antibiotics, serum or antifungals to prepare the transfection mix. LNA Gapmers were added to 200 μL of the transfection mix at a final concentration of 30 nM, placed in 12-well plates and incubated for minimum 10 min at room temperature. Fibroblasts were then seeded at a density of 5 × 10^5^ cells per well in 200 μL of growth media and incubated with the transfection mixes at 37°C, 5% (v/v) CO_2_ overnight. The next day, 800 μL of media (0.1% FBS) was added to the wells to dilute the lipid-LNA complexes and reduce the toxicity of the reaction. The cells were stimulated with 3 ng/ml of IL-1β and incubated for 24 h before harvesting for RNA extraction and ELISA analysis. LNA Gapmer sequences: Negative Control LNA1—TCATACTATATGACAG; Negative Control LNA2—GACGGTAAGTAGGCGA; IL7AS LNA1—GGCGTGAGAGTAAAGC; IL7AS LNA2—GTGCTTAGGCTTAGAG; MIR3142HG LNA1**—**GTAAACGAGTAGCAGC; MIR3142HG LNA2—GAACATGGTTACGTGT.

### Measurement of IL-6, IL-8, and CCL2 Release

Supernatants of cultured lung fibroblasts were collected and used to assess secretion of IL-6 and CCL2, using the DuoSet ELISA (Enzyme-linked immunosorbent assay, DY206 and DY279) Development System Kits (R&D Systems Europe, UK) and IL-8 (Ready-SET-Go!®, eBioscience), following the manufacturer's instructions.

### Quantitative PCR Validation of lncRNA and miRNA Expression

For quantitative PCR (qPCR) of mRNA and lncRNAs, cDNA libraries were prepared from total RNA using the High capacity cDNA RT kit (Applied Biosystems, Life Technologies, 4368813). Expression of mRNAs and lncRNAs were determined by qPCR using the SYBR® Green PCR mix (Applied Biosystems; primers were obtained from Sigma-Aldrich). For analysis, the 2^−(ΔΔ*Ct*)^ method was used to determine relative-quantities of individual mRNAs and lncRNAs which were normalized to 18S ribosomal RNA. qRT-PCR primer sequences: 18S—AAACGGCTACCACATCCAAG (Forward), CCTCCAATGGATCCTCGTTA (Reverse); IL7AS—GTGGACGATGCCAAGTCGT (Forward), AGGTGCATGTACAGCAGACG (Reverse); MIR3142HG—AGCTTGGAAGACTGGAGACAG (Forward), TCACAGGAACTCACACTCCT (Reverse).

For quantitative PCR (qPCR) of miRNAs, the miScript® II RT kit (Qiagen) with HiSpec buffer was used to prepare cDNA for mature miRNA quantification following the manufacturer's instructions. The miScript SYBR® Green PCR kit (Qiagen) was used in combination with the miScript Primer Assays for the detection of miR-3142 and miR-146a. For analysis, the 2^−(ΔΔ*Ct*)^ method was used to determine relative-quantities of the miRNAs which were normalized to SNORD61 (MS00033705, Qiagen). Mature miRNA sequences (Qiagen): 5′AAGGCCUUUCUGAACCUUCAGA (miR-3142), 5′UGAGAACUGAAUUCCAUGGGUU (miR-146a).

### Transcriptome Analysis of IL-1β-Stimulated Lung Fibroblasts

Total RNA was extracted from lung fibroblast exposed to either buffer (controls) or 3 ng/ml of IL-1β for 6 h. Paired-end and stranded 75bp sequencing data was obtained using the Illumina HiSeq4000 at the Oxford Genomics Center at the Wellcome Center for Human Genetics (funded by Wellcome Trust grant reference 203141/Z/16/Z). The paired end reads were aligned to the human reference genome (hg38) using Hisat2 (version 2.0.4) (26, 27) using the following command line options: hisat2 -q –dta –rna-strandness FR –x <reference-genone.gtf> −1 <forward_strand.fa> −2 <reverse-strand file.fa> –S <output.sam>. Output SAM files were then sorted and converted to BAM files (samtools sort –@ 8 –o output.bam output.sam) and indexed (samtools index –b output.bam) in Samtools (28). The profile of gene expression [using the Gencode v27 database and additional novel lncRNA (25)] in the BAM files for each samples were determined using Stringtie (29): stringtie <sample.BAM> –G <Gencodev27.gtf> –o <samples.gtf> –e –A <sample.txt>. The differential expression of gene derived from Gencode v27 and our recently generated list of novel lncRNA implicated in the innate immune response (25) was assessed with the geometric option in Cuffdiff v2.2.1.3 [part of the Cufflinks suite (30)] using a significance threshold of *q* (FDR) < 0.05. The command line options were as follows: cuffdiff –FDR = 0.05 –min-alignment-count = 10 –library-norm-method = geometric –dispersion-method = pooled -u <reference_genome.gtf> <control_1.bam>, <control_x.bam> <activated_1.bam>, <activated_x.bam> -o <output_file_name>.

### Principle Component Analysis and Hierarchical Clustering

The abundance of Gencode v27 defined genes in individual samples was defined as the fragments per kilobase exon per million reads mapped (FPKM) and determined using Stringtie (see above). PCA and hierarchical clustering on Gencode v27 protein coding genes demonstrating an expression >1 FPKM was performed using Genesis (v1.7.7) (31). Data was log2 transformed following the addition of 1 FPKM. The threshold for reporting gene expression at FPKM >1 is based upon the ability to validate sequencing data using qRT-PCR.

### Data Access

RNA sequencing data for control and IL1β-stimulated fibroblasts can be obtained at the Gene Expression Omnibus at GSE121241.

## Results

### Differential Expression of Protein-Coding Genes in IL-1β-Stimulated Lung Fibroblasts

**Figure 1:**
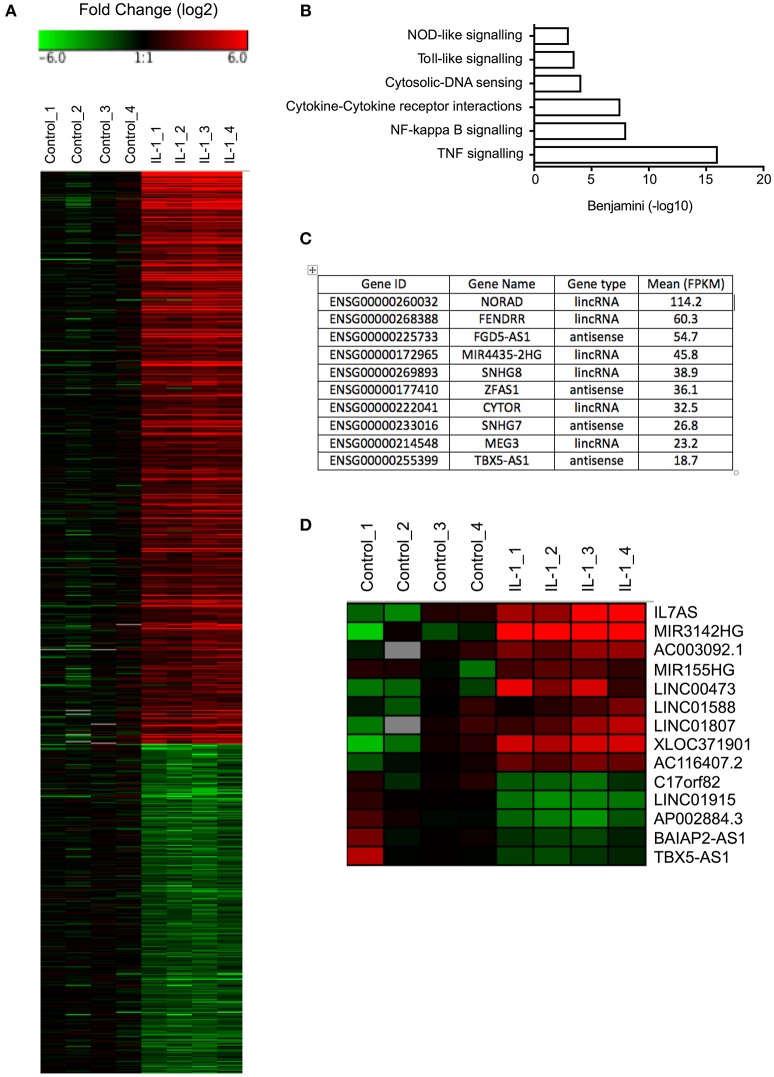
Differential expression of mRNAs and lncRNAs following IL-1β-stimulation of control lung fibroblasts**. (A)** Heat map showing the differential expression of mRNAs in control fibroblasts following IL-1β stimulation for 6 h. **(B)** Pathway analysis of up-regulated mRNAs. **(C)** Top 10 most highly expressed lncRNA in non-stimulated control fibroblasts. **(D)** Heat map showing the differential expression of lncRNAs in control fibroblasts following IL-1β stimulation for 6 h.

Initial studies were undertaken to examine the IL-1β-induced changes in mRNA expression in control fibroblasts (Table S1). Selecting only those mRNAs showing a fold-change >2, absolute change of 1 FPKM and FDR < 0.05, we identified 453 up-regulated and 261 down-regulated mRNAs (Figure 1A). As might be expected, pathway analysis (DAVID Bioinformatics resources 6.8; https://david.ncifcrf.gov/home.jsp) showed that the up-regulated mRNAs were associated with multiple inflammatory pathways (Figure 1B). No pathways were highlighted with the down-regulated mRNAs.

### Profile of Primary miRNA and lncRNAs Expression in Control Lung Fibroblasts

Before investigating those lncRNAs that were differentially expressed in response to IL-1β, we initially examined the profile of lncRNA expression in non-stimulated control fibroblasts. As a result of the difficulty in assigning sequencing data to either the original mRNA or pseudogenes during alignment, the pseudogenes were excluded from this analysis. Using a cut-off of FPKM > 1 to identify those expressed at physiologically relevant levels, we identified 484 lncRNAs that could be divided into 225 lincRNAs and 259 antisense (Table S2). Amongst the most highly expressed lncRNAs were NORAD (Non-coding RNA activated by DNA damage), a lncRNA that binds Pumilo proteins and regulates genomic stability (32, 33), MIR4435-2HG, a host gene (primary miRNA) for miR-4435, two small nucleolar host genes (SNHG7/8) and FENDRR (FOXF1 adjacent non-coding developmental regulatory RNA) a lncRNA involved in heart and body wall development (34) (Figure 1C).

### Differential Expression of Long Non-coding RNAs in IL-1β-Stimulated Control Lung Fibroblasts

To identify those lncRNAs that might mediate the inflammatory response, we compared the profile of lncRNA expression in control and IL-1β-stimulated fibroblasts at 6 h. Using the same criteria as was applied to mRNAs (FDR < 0.05, fold change >2 and absolute expression change >1 FPKM), we showed differential expression of 12 lincRNAs and 2 antisense, of which 7 were up-regulated and 5 down-regulated (Figure 1D). Of these, IL7AS and MIR3142HG showed the largest fold-changes [IL7AS (48-fold) and MIR3142HG (157-fold)] and absolute-change [IL7AS (9.2 FPKM) and MIR3142HG (5.5 FPKM)] (Table S3).

### IL7AS and MIR3142HG Regulate the IL-1β-Induced Inflammatory Response in Control Lung Fibroblasts

**Figure 2:**
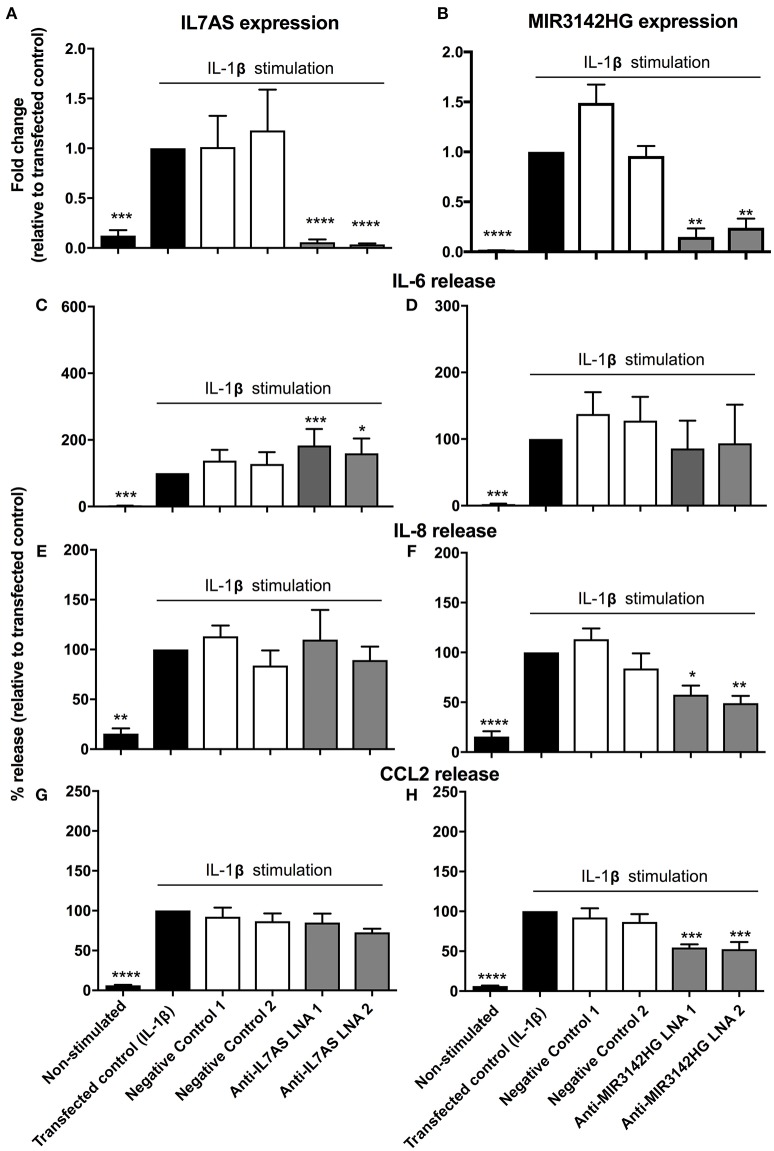
IL7AS and MIR3142HG regulate the IL-1β-stimulated inflammatory response in control fibroblasts. Control fibroblasts were transfected overnight with LNA antisense sequences against IL7AS (A/C/E/G) and MIR3142HG (B/D/F/H) or scrambled (negative) controls. Cell were then stimulated with IL-1β for 24 h prior to isolation of RNA and measurement of IL7AS **(A)** or MIR3142HG **(B)** by qRT-PCR or measurement of supernatant IL-6 **(C,D)**, IL-8 **(E,F)**, and CCL2 **(G,H)** by ELISA. Data represents the mean ± SEM of five control individuals. Following normalization against the IL1β-stimulated cells (100%), statistical significance was assessed (vs. IL1β-stimulated cells) using the repeat measures 1-way analysis of variance (ANOVA) with a Dunnett's test where *p < 0.05, **p < 0.01, ***p < 0.001, and ****p < 0.0001.

In subsequent studies we investigated the function of IL7AS and MIR3142HG in lung fibroblasts during the IL-1β-induced inflammatory response. To this end, we identified 2 locked nucleic acid based (LNA) antisense sequences against IL7AS (Figure 2A) and MIR3142HG (Figure 2B) that produced 50–85% knockdown following overnight transfection into fibroblasts and stimulation with IL-1β for 24 h. Following exposure to IL-1β, we observed increased release of the inflammatory mediators IL-6 (Figures 2C,D), IL-8 (Figures 2E,F), and CCL2 (Figures 2G,H). Knockdown of IL7AS enhanced the release of IL-6 (Figure 2C) but had no effect upon IL-8 or CCL2 (Figures 2E,G). In contrast, MIR3142HG had no effect upon IL-6 (Figure 2D) but significantly reduced the release of IL-8 and CCL2 (Figures 2F,H). This indicates that IL7AS and MIR3142HG differentially regulate the release of inflammatory mediators during IL-1β-induced activation, with IL7AS being a negative regulator of IL6 release and MIR3142HG a positive regulator of IL8 and CCL2 release.

### IL7AS and MIR3142HG and the IL-1β-Induced Inflammatory Response in IPF Fibroblasts

**Figure 3:**
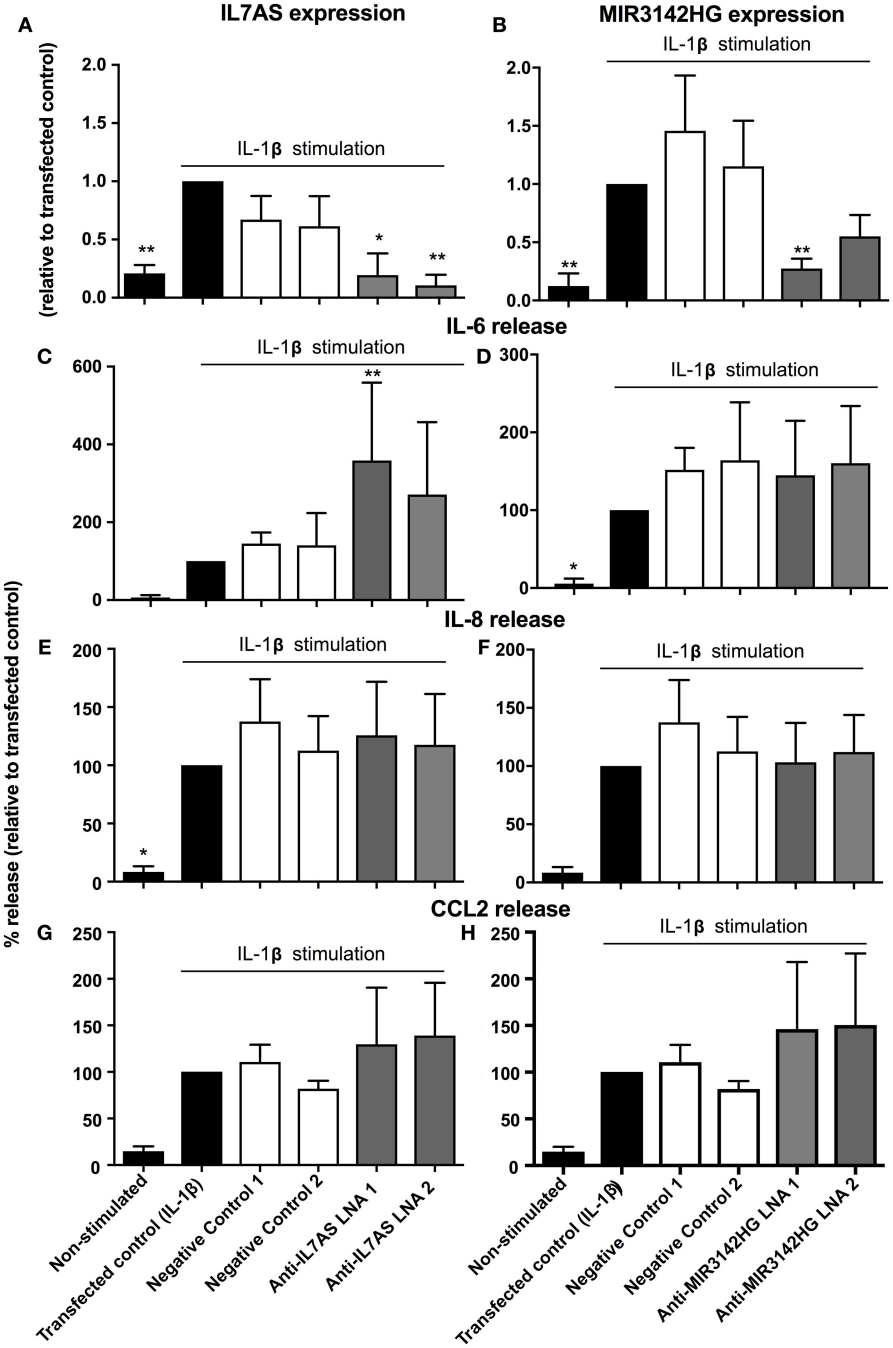
IL7AS but not MIR3142HG regulates the IL-β-stimulated inflammatory response in IPF fibroblasts. IPF fibroblasts were transfected overnight with LNA antisense sequences against IL7AS **(A,C,E,G)** and MIR3142HG **(B,D,F,H)** or scrambled (negative) controls. Cell were then stimulated with IL-1β for 24 h prior to isolation of RNA and measurement of IL7AS **(A)** or MIR3142HG **(B)** by qRT-PCR or measurement of supernatant IL-6 **(C,D)**, IL-8 **(E,F)**, and CCL2 **(G,H)** by ELISA. Data represents the mean ± SEM of five IPF individuals. Following normalization against the IL1β-stimulated cells (100%), statistical significance was assessed (vs. IL1β-stimulated cells) using the repeat measures 1-way analysis of variance (ANOVA) with a Dunnett's test where *p < 0.05 and **p < 0.01.

Previous studies have demonstrated differences in the phenotypic responses between lung fibroblasts derived from control and IPF patients including a recent meta-analysis of microarray data showing repression of inflammation and immune pathways in IPF (35). We therefore decided to examine whether IL7AS and MIR3142HG have comparable functions during the IL1β-induced inflammatory response in IPF lung fibroblasts. Using LNA-based antisense, we once again demonstrated 50–85% knockdown of IL7AS (Figure 3A) and MIR3142HG (Figure 3B) following overnight transfection into IPF fibroblasts and stimulation with IL-1β for 24 h. As with control cells, knockdown of IL7AS caused a significant increase in IL-6 release (Figure 3C) but had no effect upon IL-8 (Figure 3E) or CCL2 production (Figure 3G). Interestingly, MIR3142HG knockdown had no effect upon release of either IL-6, IL-8, or CCL2 (Figures 3D,F,H). These results therefore indicated differences in the function of MIR3142HG but not IL7AS in the IL1β-induced inflammatory responses between control and IPF fibroblasts.

### IPF Fibroblasts Showed Reduced IL-1β-Induced MIR3142HG/miR-146a Production and Inflammatory Response

**Figure 4:**
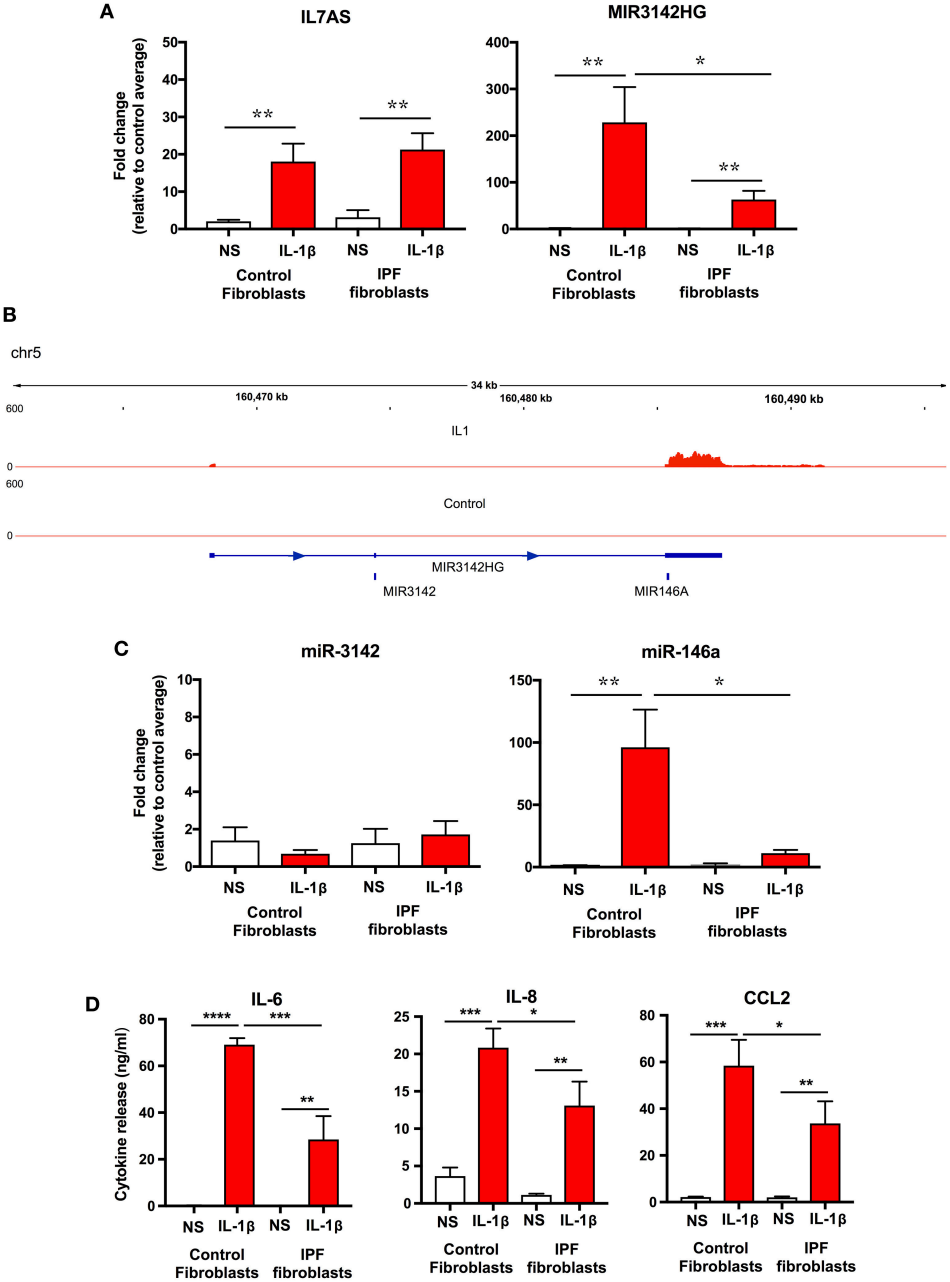
IL-1β-induced expression of IL7AS, MIR3142HG, miR-146a, miR-3142 and inflammatory mediators in control and IPF fibroblasts**. (A)** Control and IPF fibroblasts were incubated in the absence of presence of IL-1β for 24 h and the fold-change in the IL7AS and MIR3142 expression determined by qRT-PCR, **(B)** Aligned sequencing data (merged BAM files) showing MIR3142HG from control and IL-1β-stimulated control fibroblasts was visualized using the IGV genome browser (https://software.broadinstitute.org/software/igv/). Control and IPF fibroblasts were incubated in the absence of presence of IL-1β for 24 h before determination of the fold-change in miR-3142 and miR-146a expression by qRT-PCR **(C)** and the release of IL-6, IL-8, and CCL2 by ELISA **(D)**. Values are the mean ± SEM of five control and IPF patients and statistical significance was assessed using 1-way analysis of variance (ANOVA) where *p < 0.05, **p < 0.01, ***p < 0.001, and ****p < 0.0001.

To examine whether the differences in lncRNA function might be related to changes in expression, initial comparison of raw dCT values obtained from qRT-PCR showed no difference in the baseline expression of IL7AS (Control = 23.3 ± 1.0; IPF = 22.2 ± 1.1) and MIR3142HG (Control = 26.35 ± 0.7; IPF = 27.8 ± 0.5) between control and IPF fibroblasts. However, although there was no significant difference in the IL-1β-induced IL7AS expression between control and IPF cells, there was a reduction in the MIR3142HG production in IPF (Figure 4A). Thus, we observed a ~220-fold increase in MIR3142HG expression in control fibroblasts following exposure to IL-1β, compared with an ~50-fold increase in IPF fibroblasts. Although MIR3142HG is the host gene for miR-3142, this also contains miR-146a, a widely reported regulator of inflammation and the immune response (Figure 4B) (11, 12). Examination of the histogram peaks produced by our RNA sequencing data for MIR3142HG (using the IGV genome browser) indicated that this may be processed to produce miR-146a and not miR-3142 (Figure 4B). In support of this contention, we showed a significant ~100-fold increase in miR-146a but not miR-3142 (Figure 4C) following IL-1β stimulation of control fibroblasts. Interestingly, as with MIR3142HG, we observed a significant reduction in miR-146a expression in IL-1β-stimulated IPF fibroblasts (Figure 4C), providing additional evidence to support the metabolism of MIR3142HG to miR-146a. Given our previous data showing that MIR3142HG (and potentially miR-146a) is a positive regulator of the inflammatory response (Figure 2), it might be expected that the reduced MIR3142HG/miR-146a production in IPF fibroblasts would result in a diminished cytokine release. In support of this contention, we demonstrated that IL-1β-induced IL-6, IL-8, and CCL2 release was significantly attenuated in IPF compared with control fibroblasts (Figure 4D). In summary, these studies show a reduced production of MIR3142HG and miR-146a in IL-1β stimulated IPF fibroblasts and suggest that this is responsible for the reduced cytokine production.

To ascertain whether IL7AS and MIR3142HG are also upregulated in IPF lung *in situ*, we analyzed RNA sequencing data of biopsy samples obtained from control (*n* = 19) and IPF (*n* = 20) lungs (Table S4) (36). However, we were unable to demonstrate significant changes in IL7AS and MIR3142HG although this could be related to the fact that these biopsies represent a mixed cell population and/or that these lncRNAs are only induced in a highly inflammatory environment i.e., following exposure to IL-1β. Interestingly, we did observe an ~2-fold increase in IL-1β expression in IPF vs. control biopsies.

### IL-1β-Induced MIR3142HG/IL7AS Production and the Inflammatory Response Are Mediated via NF-κB

**Figure 5:**
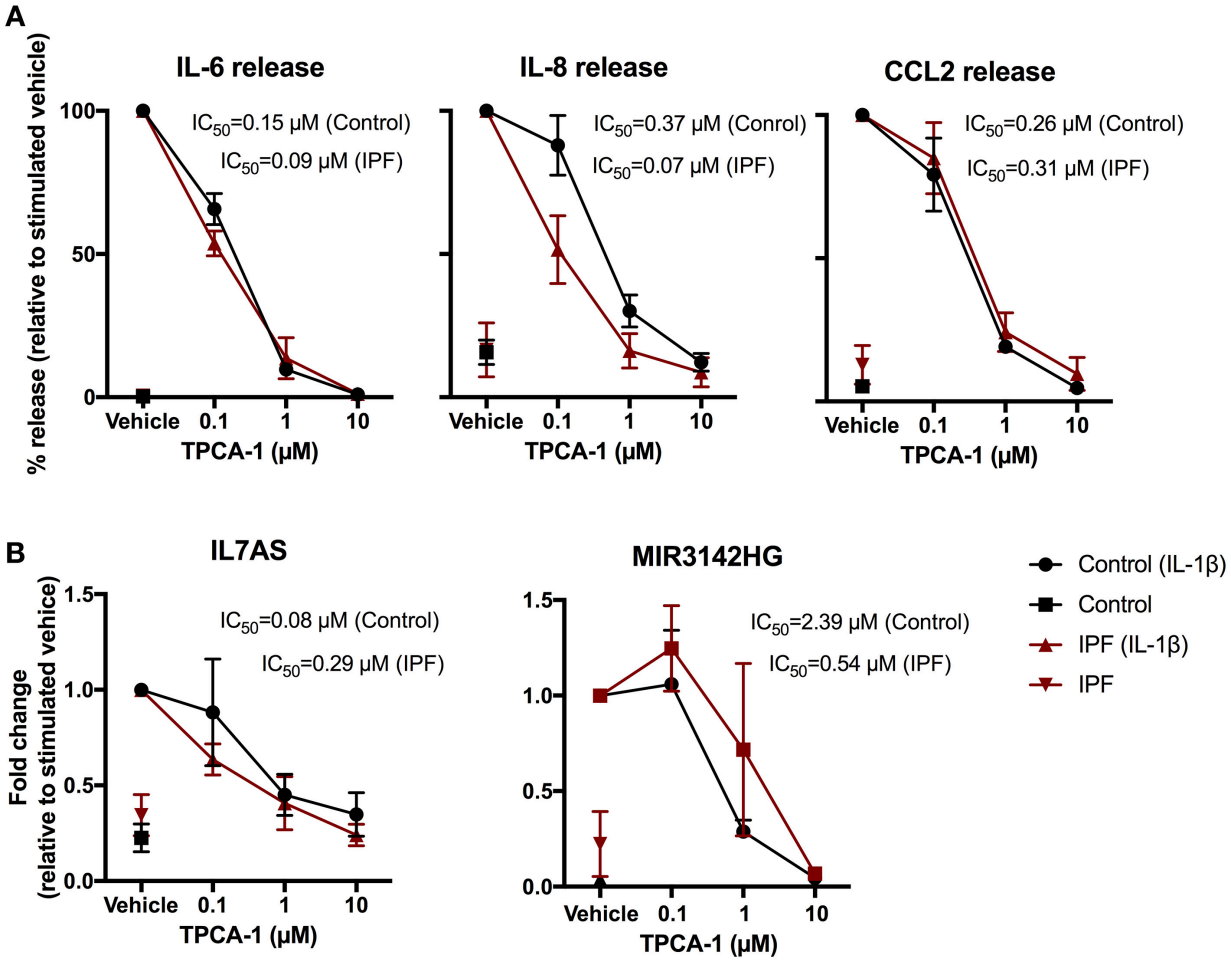
IL-1β-induced expression of IL7AS, MIR3142HG, and the inflammatory mediators is mediated via the NF-κB signaling pathway in control and IPF fibroblasts. Control and IPF fibroblasts were pre-incubated in the stated concentration of TPCA-1 or 0.1% (v/v) DMSO (vehicle) and then incubated in absence or presence of IL-1β for 24 h prior to measurement of IL6, IL8 and CCL2 release **(A)** and IL7AS and MIR3142HG expression **(B)**. Data is normalized against IL-1β stimulated cells (100%) and represents the mean ± SEM of five control and IPF patients. The logIC_50_ for each individual was determined in GraphPad Prism and comparison between control and IPF groups was performed using an unpaired *t*-test. The IC_50_ was calculated from the mean logIC_50_ values.

To elucidate the intracellular pathways that regulate the IL-1β induced responses, we adopted a pharmacological approach to investigate the role of nuclear factor-kappa B (NF-κB) signaling pathway. To this end, we examined the action of an inhibitor of IKK2 (TPCA-1) (37), an upstream activator of NF-κB. Exposure to TPCA-1 resulted in a concentration dependent reduction in IL-1β-induced IL-6, IL-8, and CCL2 release from control fibroblasts with IC_50_ of 0.15, 0.37, and 0.26 μM, respectively (Figure 5A). This was not significantly different from the inhibition observed in IPF fibroblasts (IL6–0.09 μM, IL-8–0.07 μM, and CCL2–0.31 μM). In all cases, complete attenuation of cytokine release was observed at 10 μM TPCA-1 (Figure 5A). TPCA-1 also resulted in a concentration dependent inhibition of IL7AS and MIR3142HG generation, with no significant difference between control and IPF fibroblasts Figure 5B. These studies indicate that the production of IL7AS, MIR3142HG, and the inflammatory cytokines is mediated via an NF-κB pathway.

## Discussion

Whilst the role of inflammation in the initiation and progression of the IPF remains unclear, there is evidence to indicate that IPF is associated with inflammation and changes in the innate and adaptive immune response (38, 39). Specifically, the secretion of the pro-inflammatory cytokine IL-1β has been linked to the progression and development of fibrosis by enhancing the expression of the inflammatory mediators interleukin 6 (IL-6) and tumor necrosis factor α (TNF-α), disrupting alveolar architecture and by increasing pulmonary fibroblasts and collagen deposition (40). Release of the pro-fibrotic cytokines TGF-β1 and PDGF may also be stimulated by IL-1β in bronchoalveolar lavage (BAL) fluid. IL-1β was also shown to increase the infiltration of neutrophils and macrophages to the lungs (40, 41) and elevate the expression of matrix metalloproteinases (MMPs) MMP-9/12 and chemokine (C-X-C motif) ligands (CXCL) CXCL1/2 (41). Given the potential importance of IL-1β both in the induction of inflammation and the development of IPF, we have for the first time examined the role of lncRNAs in the IL-1β-induced inflammatory response of control human lung fibroblasts and compared this with fibroblasts obtained from patients with IPF.

Initial studies cataloged those lncRNAs that are changed following IL-1β-stimulation and demonstrated differential expression of 14 lncRNAs. This included IL7AS, a syntenically conserved antisense that overlaps and is expressed in a bi-directional manner with the promoter of IL-7 (25), as well as MIR3142HG, the host gene for miR-3142 and miR-146a. In subsequent functional studies, we employed LNA antisense knockdown to examine the role of IL7AS and MIR3142HG, the two most highly induced lncRNAs, during the IL-1β-induced inflammatory response. As we have previously reported (25, 42), IL7AS knockdown was shown to increase IL-6 release from both control and IPF-derived fibroblasts, indicating that this is a negative regulator. However, in contrast to these earlier reports, this action appear to be selective for IL-6, as there was no effect upon the release of IL-8 and CCL2 (25, 42). Given that lncRNA expression and function is thought to be highly cell-type specific (43), our demonstration that IL7AS also regulates the inflammatory response in lung fibroblasts provides additional evidence to indicate that IL7AS is a central regulator of inflammation.

In contrast to IL7AS, MIR3142HG knockdown showed that this lncRNA was a positive regulator of IL-8 and CCL2 release. Subsequent analysis of miRNA expression indicated that MIR3142HG was metabolized to miR-146a but not miR-3142, which could mean that the biological actions of MIR3142HG are secondary to the production of miR-146a. Unfortunately, it is not possible to separate the actions of MIR3142HG from those of miRNA-146a since miRNA knockdown would also be expected to reduce the levels of the host gene. However, miR-146a is a well-characterized miRNA that has previously been reported to negatively regulate the inflammatory response through down-regulation of TRAF6 and IRAK1 (44, 45). This would therefore suggest that MIR3142HG is the biologically active molecule or, if mediated by miR-146a, that this miRNA has a different function and mechanism of action in human lung fibroblasts.

Subsequent comparison with IPF fibroblasts showed no changes in IL-1β-induced IL7AS production but a significant reduction in the generation of MIR3142HG and miR-146a, which correlated with a decrease in IL-6, IL-8, and CCL2 release. Given that MIR3142HG knockdown was shown to significantly reduce IL-8 and CCL2 release, and to partially reduce IL-6 release in control fibroblasts, this indicates that the drop in MIR3142HG expression may be responsible for the reduced inflammatory response in IL-1β-stimulated IPF fibroblasts.

In conclusion, we have cataloged those lncRNAs that are differentially expressed following IL-1β-activation of human lung fibroblasts, shown that IL7AS and MIR3142HG/miR-146a regulate the inflammatory response and demonstrated that the reduced inflammatory response in IPF fibroblasts is correlated with attenuated expression of MIR3142HG/miR-146a.

## Author Contributions

MH and BR contributed to the acquisition, analysis, and interpretation of the data. CF-B, LM, and DC contributed to the design and drafting of the work. ML contributed to the concept, design, and drafting of the work.

### Conflict of Interest Statement

LM was employed by AstraZeneca and DL was employed by Medimmune and Boehringer Ingelheim Ltd. The remaining authors declare that the research was conducted in the absence of any commercial or financial relationships that could be construed as a potential conflict of interest.

## Supplementary Material

Table S1 Overall profile of gene expression in human lung fibroblasts.

Table S2 Profile of lncRNA expression in human lung fibroblasts.

Table S3 Differential expression of lncRNAs in IL-1β-stimulated human lung fibroblasts.

Table S4 Differential gene expression in control vs. IPF biopsies.

## Abbreviations

IPF: Idiopathic Pulmonary Fibrosis (IPF)
ncRNA: non-coding RNA
lncRNA: long non-coding RNA
lincRNA: long intergenic non-coding RNA
IL: interleukin
LNA: Locked nucleic acid.
